# *Ganoderma lucidum* Triterpenoids Suppress Adipogenesis and Obesity via PRKCQ Activation: An Integrated In Vivo, In Vitro, and Systems Pharmacology Study

**DOI:** 10.3390/foods15020325

**Published:** 2026-01-15

**Authors:** Boyi Li, Jianing Chen, Yuanyuan Sun, Jianping Gao, Minyan Hu, Juan Xu, Siying Wang, Na Feng, Haishun Xu, Zhiyan Jiang, Xueqian Wu, Ying Wang

**Affiliations:** 1 College of Food and Health, Zhejiang A&F University, Hangzhou 311300, China; 18731168728@163.com (B.L.); jianingchen0327@outlook.com (J.C.); yysun_oo@163.com (Y.S.); ygoyyds2021@163.com (J.G.); huminyani@163.com (M.H.); xujuanzju@163.com (J.X.); wangsiying@zafu.edu.cn (S.W.); 20160019@zafu.edu.cn (H.X.); zyjiang@zafu.edu.cn (Z.J.); 2 Institute of Edible Fungi, Shanghai Academy of Agricultural Sciences, Shanghai 201403, China; fengna006@163.com

**Keywords:** *Ganoderma lucidum* triterpenoids, obesity, adipocyte differentiation, systematic pharmacology, machine learning, PRKCQ

## Abstract

*Ganoderma lucidum* triterpenoids (GLTs) exhibit potential anti-obesity activity. However, their mechanism remains unclear. In this study, triterpenoids were extracted from *G. lucidum* via ultrahigh-pressure extraction. Using a high-fat diet (HFD)-induced mouse model, we showed that GLT treatment (100 and 200 mg/kg) significantly reduced body weight and lipid accumulation without changing food intake. Next, we found that GLT significantly inhibited preadipocyte differentiation and adipogenesis and reduced the expression of adipogenic genes, including *PPARγ*, *C/EBPα*, *FASN*, and *SCD-1.* Moreover, network pharmacology predicted a total of 306 potential targets, among which *FYN*, *PRKCQ*, *PTPRF*, *HRH1,* and *HCRTR2* were identified as the core targets via a machine learning algorithm. Interestingly, GLT upregulated the expression of PRKCQ, while the deletion of PRKCQ significantly reversed the anti-adipogenic effect of GLT. In addition, we found that neutral GLT may play a dominant role in inhibiting adipogenic differentiation. These findings suggest for the first time that GLT inhibits adipogenesis and lipid accumulation via the induction of PRKCQ in adipocytes. This study provides a scientific basis for the application of GLT in the prevention and treatment of obesity, as both a pharmaceutical agent and a functional food.

## 1. Introduction

Obesity has emerged as a growing public health concern worldwide [1,2] that increases the risk for many diseases including cardiovascular and cerebrovascular diseases, type 2 diabetes mellitus, non-alcoholic fatty liver disease, and other chronic diseases [3,4]. Data from the World Health Organization (WHO) and the World Obesity Alliance indicate that there are currently 2603 million overweight or obese people worldwide, and this is projected to reach 4005 million by 2035. As the prevalence of obesity is rapidly increasing globally, effective prevention and treatment of obesity have become urgent issues for public health.

Obesity arises from complex factors that lead to long-term metabolic dysfunction, ultimately resulting in the excessive accumulation of body fat. The accumulation of adipose tissue caused by adipocyte differentiation is the key to the occurrence of obesity [5]. Adipocyte differentiation involves the transformation of fibroblast-like preadipocytes into mature adipocytes. Therefore, inhibiting adipocyte differentiation and adipogenesis represents a promising therapeutic target for preventing and treating obesity [6]. Currently, cell culture models greatly facilitate our understanding of the regulatory processes and mechanisms of adipogenesis, such as 3T3-L1 preadipocytes which are widely used in research on adipocyte differentiation including the study of anti-obesity agents targeting adipogenic differentiation [7,8,9,10,11].

At present, traditional Chinese medicine (TCM) has shown promising anti-obesity properties by inhibiting adipocyte differentiation, for example, ginger [7], Radix bupleuri [11], Salvia miltiorrhiza Bunge, and Polygonum multiflorum Thunb [12], as reviewed previously. *G. lucidum*, an edible mushroom with medicinal and dietary properties, has been consumed medicinally for more than 2000 years to promote vitality and longevity [13]. GLT is one of the key active compounds in *G. lucidum*, primarily of the lanostane type. Its characteristic tetracyclic backbone, often substituted with hydroxyl, carboxyl, or keto groups, critically influences the bioactivity and solubility of these triterpenoids. The fruiting body and spores of *G. lucidum* serve as the primary accumulation sites for triterpenoids. GLT has demonstrated various pharmacological effects such as anti-tumor, anti-inflammation, anti-viral, and immunomodulatory effects [14]. Several studies have reported the anti-lipogenic effects of triterpenoids extracted from other herbs such as Radix bupleuri (Saikosaponin A and D) [11], Tripterygium Wilfordi (Celastrol) [15], and ginseng (Ginsenoside Rb1) [16]. At present, studies reporting the relationship between Ganoderma triterpenoids and adipocyte differentiation are scarce. For example, Su et al. found that the triterpenoid ganodapplanoic acid I, extracted from *Ganoderma*, effectively inhibited fat formation by downregulating the expression of major proteins (FASN, PPARγ, and CEBPβ) involved in differentiation and fat formation in 3T3-L1 adipocytes [17]. However, the underlying molecular mechanisms by which GLT inhibits adipogenic differentiation remain largely unknown.

TCM is defined as having multi-component, multi-target, and multi-pathway characteristics. For instance, *G. lucidum* is valued in TCM for its complex mixture of compounds (polysaccharides, triterpenoids, and sterols) interacting with multiple biological targets (e.g., CASP3, TNF-α, ACOX1, and GDF15) and pathways (e.g., MAPK, Akt, NF-κB, and Nrf2/HO-1) [13]. Systems pharmacology is an emerging field that integrates classical pharmacology, bioinformatics, computer science, and network pharmacology. It offers a novel methodology for investigating complex systems within TCM [18]. To date, many studies have used systems pharmacology, especially network pharmacology and molecular docking, to perform active ingredient–disease target analysis of TCM [19,20]. However, systems pharmacology studies of GLT against obesity have not been reported.

Therefore, the present study aims to investigate the anti-obesity effects and underlying molecular mechanisms of GLT. The UPLC-QQQ-MS/MS method was used to analyze the composition and content of GLT; network pharmacology in combination with machine learning was used to identify the potential activity components and targets; and both an HFD-induced obese mouse model and 3T3-L1 cell model were employed to verify the anti-lipogenic effects as well as the identified targets. Our study may provide a scientific basis for using GLT as a promising candidate in preventing and treating obesity.

## 2. Materials and Methods

### 2.1. Chemicals and Reagents

Dulbecco’s modified Eagle’s medium (DMEM), penicillin-streptomycin solution, and fetal bovine serum (FBS) were obtained from Gibco (Grand Island, NY, USA). 3-(4, 5-dimethylthia-zol-2-yl)-2, 5-diphenyltetrazolium bromide (MTT) was purchased from AMRESCO (Solon, OH, USA). 3-isobutyl-1-methylxanthine (IBMX), dexamethasone (DEX), 4, 6-Diamidino-2-phenylindole (DAPI), and insulin were commercially acquired from Sigma-Aldrich (St. Louis, MO, USA). Oil Red O was obtained from Solarbio Science & Technology (Beijing, China). Bodipy 493/503 was acquired from Thermo Fisher Scientific (Waltham, MA, USA). The triglyceride (TG) and total cholesterol (TC) enzymatic assay kit was purchased from Nanjing Jiancheng Bioengineering institute (Nanjing, China). The bicinchoninic acid (BCA) protein assay kit was acquired from Pierce (Rockford, IL, USA). The PRKCQ (#AF6394) antibody was sourced from Affinity Biosciences (Cincinnati, OH, USA). The polyclonal β-actin (#4967) and horseradish peroxidase-conjugated anti-rabbit secondary antibodies were obtained from Cell Signaling Technology (Danvers, MA, USA). The enhanced chemiluminescence Substrate (Western Lightning^TM^ Plus-ECL) was obtained from Perkin-Elmer Inc. (Waltham, MA, USA). The low-fat diet (LFD) with 10% calories from fat (D12450) and the high-fat diet (HFD) with 60% calories from fat (D12492) were obtained from Research Diet (New Brunswick, NJ, USA).

### 2.2. Sample Preparation and Triterpenoid Content Detection

The *G. lucidum* fruiting body was obtained from Zhejiang Wuyangtang Pharmaceutical Co., Ltd. (Zhejiang, Lishui, China). Ultrahigh-pressure extraction was used to obtain triterpenoids from the *G. lucidum* fruiting body. Briefly, the optimized parameters for ultrahigh-pressure-assisted extraction were determined as follows: pressure of 350 MPa, solid–liquid ratio of 1:20 g/mL, holding time of 7 min, and ethanol concentration of 90%. The samples in the high-pressure equipment were removed and centrifuged at 10,000 rpm for 10 min to isolate the supernatant. The triterpenoid content was detected using the vanillin-perchloric acid chromogenic method as previously described [21]. Oleanolic acid was used as the standard to calculate the concentration.

### 2.3. UPLC-QQQ-MS/MS Analysis

The composition of *G. lucidum* triterpenoids extract was analyzed using ultra-high- performance liquid chromatography triple quadrupole tandem mass spectrometry (UPLC-QQQ-MS/MS) with reference to the analytical method described in the patents ZL202011236775.0 and ZL2021112100687.8. In brief, the compounds in *G. lucidum* substrates were isolated and purified using ultra-performance liquid chromatography (UPLC). Then, the isolated compounds were converted to the ionic state using atmospheric pressure chemical ionization (APCI) or electrospray ionization (ESI). Finally, the ionized compounds were introduced into a triple-quadrupole mass spectrometer (QQQ-MS/MS) for mass spectrometry and quantitative analysis using techniques such as multistage mass spectrometry scanning and collision-induced dissociation (CID).

#### 2.3.1. Acid Triterpenes Analysis Conditions

Chromatographic conditions: UPLC analysis was carried out on an Agilent Eclipse Plus C18 column (1.8 μm, 2.1 × 150 mm, Agilent Technologies Inc., Santa Clara, CA, USA). The detection wavelength was 254 nm, the column temperature 35 °C, the injection volume 2 μL, and the flow rate 0.4 mL/min. The mobile phase comprised (A) 0.01% acetic acid in water and (B) acetonitrile. The gradient elution program was as follows: 0 min, 74% A, 26% B; 18 min, 73% A, 27% B; 28 min, 65% A, 35% B; 31 min, 40% A, 60% B; 36 min, 10% A, 90% B; 40 min, 0% A, 100% B; 44 min, 74% A, 26% B. Mass spectrometric conditions: An electrospray ionization source with an AJS-ESI interface was used as the ion source. Detection was performed in negative-ion mode with dynamic multiple reaction monitoring (DMRM). The capillary voltage was 3500 V and the capillary outlet voltage 380 V. The dry gas flow rate was 16 L/min at a temperature of 200 °C. The sheath gas temperature was 320 °C with a flow rate of 12 L/min, and the nozzle voltage was set to 2000 V.

#### 2.3.2. Neutral Triterpenes Analysis Conditions

The chromatographic conditions for the separation of neutral triterpenes using ultra-performance liquid chromatography were as follows: UPLC was employed using an Agilent ZORBAX SB Aq column (1.8 μm, 2.1 × 150 mm, Agilent Technologies Inc., Santa Clara, CA, USA). The detection wavelength was set at 240 nm, the column temperature at 35 °C, and the injection volume at 2 μL. The flow rate was 0.4 mL/min. The mobile phase consisted of (A) 0.01% acetic acid in water and (B) methanol. The gradient elution program was as follows: 0 min, 20% A, 80% B; 8 min, 20% A, 80% B; 10 min, 10% A, 90% B; 15 min, 0% A, 100% B; 18 min, 0% A, 100% B. Mass spectrometric conditions: APCI source was used as the ion source. Detection was performed in positive-ion mode with DMRM. The capillary voltage was 3500 V, the capillary outlet voltage 380 V, and the corona needle current 8 μA. The dry gas temperature was 290 °C with a flow rate of 13 L/min. The vaporizer temperature was set to 350 °C, and the nebulizer pressure was 30 psi.

### 2.4. Animal Study

All experiments were performed following the National Institutes of Health Guide for the care and Use of Laboratory Animals. This study was authorized according to the Ethics Committee of Zhejiang A&F University (Permit Number: SYXK 2023-0015). Seven-week-old male C57BL/6J mice, obtained from SLAC (Shanghai laboratory animal center), were randomly divided into four groups (n = 9 per group): LFD (control group), HFD, low dose of GLT in HFD (HFD-100 mg/kg), and high dose of GLT in HFD (HFD-200 mg/kg). The doses of GLT (100 or 200 mg/kg) were chosen based on our previous study [22]. The mice in each group were housed three per cage (three cages per group) under specific pathogen-free conditions, with a temperature of 25–28 °C and 12/12-h light/dark cycle. GLT (dissolved in saline containing 5% Tween 80) was administered to the mice by daily gavage over a period of 12 weeks. The body weight and food intake were measured weekly. At the end of the experiment, all animals were euthanized using CO_2_ asphyxiation, and serum samples along with epididymal white adipose tissue (eWAT) were collected.

### 2.5. Histopathology Analysis

Following collection, eWAT tissues were immersion-fixed in 4% formaldehyde and then processed for paraffin embedding. 4 µm-thick sections were prepared from the tissues. These sections were then subjected to hematoxylin and eosin (H&E) staining for subsequent histopathological evaluation.

### 2.6. Network Pharmacology

#### 2.6.1. Prediction Targets of GLT for the Regulation of Adipogenesis

The TCMSP database (http://tcmspnw.com/), SwissTarget Prediction database (http://swisstargetprediction.ch/), as well as PharmMapper database (http://www.lilab-ecust.cn/pharmmapper/) were used to predict the potential targets of the main components of GLT. The keyword “adipocyte differentiation” was searched in the GeneCards database (https://www.genecards.org/) and OMIM database (http://www.omim.org/) to obtain the disease targets related to obesity. The potential targets of GLT for regulating adipocyte differentiation were obtained after intersection using a Venn diagram (https://www.bioinformatics.com.cn). Cytoscape 3.9.1 was used to analyzed the network of “drug–active ingredient–disease target”, and the plug-in CytoNCA was used to calculate the “degree centrality (DC)”.

#### 2.6.2. Differential Expression Analysis of the Selected Potential Targets Between Healthy Person and Patients with Obesity

The publicly available expression profile of the gene array was retrieved from the GEO database (http://www.ncbi.nlm.nih.gov/geo/). The expression of the selected potential targets in Section 2.6.1 was analyzed in the visceral adipose tissue of patients with obesity (GEO accession number: GSE24883). A heatmap was built using the pheatmap package in R software 4.4.3, and the differentially expressed targets were used for subsequent analysis.

#### 2.6.3. Screening of Core Target

LASSO regression and SVM-RFE, two machine learning algorithms, were used for core target screening. LASSO can simultaneously perform feature selection and model regularization, effectively enhancing the interpretability of the model and ensuring that the selected genes are pertinent to the study aims. SVM-RFE is used to further optimize the screened genes, which recursively removes the features with the smallest weights to gradually select the most core genes. The intersection of the two screening results was the key targets. Then an ROC curve was built using the “glmnet” and “pROC” packages in R to evaluate the diagnostic value of key targets and finally identify core targets.

### 2.7. Cell Culture, Differentiation, and Treatment

The 3T3-L1 murine preadipocyte cell line was purchased from the American Type Culture Collection (ATCC, Manassas, VA, USA). Cells were cultured in DMEM containing 10% FBS and maintained in an incubator at 37 °C. At two days post-confluence (day 0, D0), the 3T3-L1 cells were differentiated using a standard differentiation medium I (DMI) containing DEX (1 μM), IBMX (0.5 mM), and insulin (1 μg/mL) for two days. Next, differentiation medium II (DMII) containing 1 μg/mL insulin was replaced every two days for maintenance differentiation treatment. To examine the effect of GLT on differentiation, the 3T3-L1 cells were treated with GLT at a concentration of 0, 50, 100, 200, or 400 μg/mL during the first two days with DMI. The extent of differentiation was examined on day 8 (D8).

### 2.8. Cell Viability Assay

An MTT assay was used to determine the effect of GLT on cell viability. Briefly, after seeding in 96-well plates (1 × 10^4^ cells/well), 3T3-L1 cells were maintained at 37 °C in DMEM with 10% FBS until reached 50% confluence. Cells were then exposed to varying concentrations of GLT (0, 50, 100, 200, 400, or 800 μg/mL) for 24 and 48 h. Afterward, cells were treated with 5 mg/mL of MTT and incubation continued at 37 °C for 4 h. After removing the supernatants, the formazan crystals were dissolved in 150 μL of dimethyl sulfoxide (DMSO) with gentle shaking for 10 min, and the absorbance was read at 490 nm.

### 2.9. Oil Red O Staining

Lipid accumulation was determined using Oil Red O staining. Briefly, 3T3-L1 cells were plated onto 6-well plates (1 × 10^5^ cells/well) and induced to differentiate, then treated with different concentrations of GLT (0, 50, 100, 200, or 400 μg/mL). After differentiation at D8, the cells were washed twice with PBS and fixed with 4% paraformaldehyde for 10 min. Subsequently, the cells were subjected to staining with Oil Red O solution (Oil Red O dye/double-distilled water = 3:2) for 40 min, washed thrice with 60% isopropanol, and imaged under a microscope.

### 2.10. Bodipy 493/503 Fluorescence Staining

At a density of 2 × 10^4^ cells/well, 3T3-L1 cells were plated into the laser confocal culture dish. After differentiation at D8, the cells were rinsed with PBS and fixed with 4% paraformaldehyde for 10 min, stained with 1 μM Bodipy 493/503 for 30 min away from light, and washed thrice with PBS. The nucleus was stained with DAPI for 15 min. After washing with PBS, the cells were observed under a confocal laser microscope (LSM880, Carl Zeiss, Oberkochen, Germany).

### 2.11. Determination of TG and TC Content

Serum was collected to measure TC and TG using an enzymatic assay kit according to the manufacturer’s instruction. 3T3-L1 cells were treated with GLT, and both the cells and medium were collected after differentiation. The GPO-PAP enzyme kit was used to determine extracellular and intracellular TG contents by following the manufacturer’s instruction. The BCA assay was employed to quantify the total cellular protein content, and the extracellular and intracellular TG content was normalized to the total protein concentration in the cell lysates.

### 2.12. RNA Extraction and qRT-PCR Analysis

Total RNA was isolated from 3T3-L1 cells with the RNA extraction kit according to the manufacturer’s protocol. Following the manufacturer’s instructions, cDNA was generated from 1 µg of total RNA using the iScript cDNA Synthesis Kit. qRT-PCR was conducted using SYBR Green Master Mix and the CFX96 Real-time PCR System (Bio-rad, Hercules, CA, USA). Using β-actin as the housekeeping gene, the relative mRNA expression levels were calculated using 2^−ΔΔCT^. The primer sequences used in this study are as follows. *PPARγ*: forward, 5′-TGTCGGTTTCAGAAGTGCCTTG-3′; reverse, 5′-TTCAGCTGGTCGATATCACTGGAG-3′. *C/EBPα*: forward, 5′-CAAGAACAGCAACG AGTACCG-3′; reverse, 5′-GTCACTCGTCAACTCCAGCAC-3′. *FASN*: forward, 5′-GGAGGTGGTGATAGCCGGTAT-3′; reverse, 5′-TGGGTAATCCATAGAGCCCAG-3′. *SCD-1*: forward, 5′-GGCTAGCTATCTCTGCGCTC-3′; reverse, 5′-GAACTGCGCT TGGAAACCTG-3′. *β-actin*: forward, 5′-CAGCTTCTTTGCAGCTCCTT-3′; reverse, 5′-CACGATGGAGGGGAATACAG-3.

### 2.13. Western Blot Analysis

The total protein was isolated from 3T3-L1 cells using standard methods and the protein concentration was examined using the BCA method. Protein samples (30 μg) were separated using 10% SDS-PAGE gel and then transferred onto PVDF membranes. Following blocking with 5% non-fat milk in Tris-buffered saline/Tween 20 (TBST), the membranes were incubated with primary antibodies against PRKCQ and β-actin (1:1000 dilution) overnight at 4 °C. After washing thrice with TBST, the membranes were further incubated with the secondary antibody (1:2000) for 1 h at room temperature. Signal detection was performed using the Western Lightning Plus ECL substrate, and Image J software (version 1.4.3) was employed to calculate the optical density.

### 2.14. siRNA Transfection

The small interfering RNAs (siRNAs) targeting *PRKCQ* were designed and synthesized by Shanghai Gima Pharmaceutical Technology Co., Ltd. (Shanghai, China), which has the following sequences: sense, 5′-GGAGAUGCGAAGACAAAUATT-3′ and antisense, 5′-UAUUUG UCUUCGCAUCUCCTT-3′. Briefly, 1 × 10^5^ cells/well 3T3-L1 cells were seeded in 6-well plates. When cell density reached about 50%, the cells were transfected with 100 nM PRKCQ siRNA or negative control siRNA using the Lipofectamine^®^ 2000 regent according to the manufacturer’s instructions. The efficiency of *PRKCQ* knockdown was confirmed using Western blotting analysis. Subsequently, the 3T3-L1 cells were induced to differentiate and treated with GLT (0, 200 μg/mL).

### 2.15. Statistical Analysis

Statistical analysis was conducted using SPSS software (version 23; SPSS Inc., Chicago, IL, USA). Data are presented as the mean ± SEM from three independent experiments. For multiple comparisons, One-way ANOVA was applied to assess statistical significance. A *p*-value of < 0.05 was considered to be statistically significant. GraphPad Prism 9.0 software was employed for figures preparation.

## 3. Results

### 3.1. Analysis of the Composition of Triterpenoid Extracted from G. lucidum

**Figure 1:**
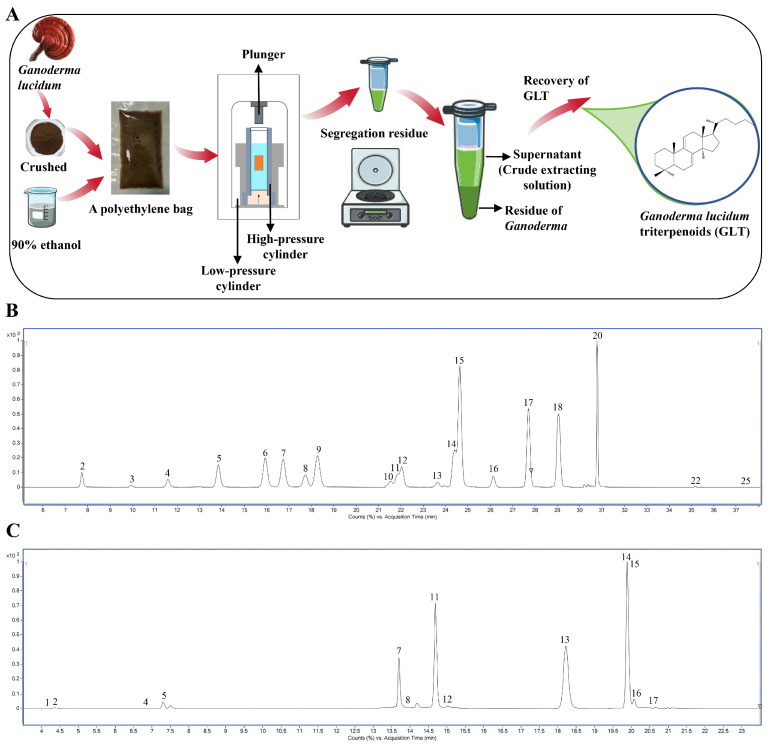
Extraction and Composition Identification of GLT. (**A**) Schematic illustration of the ultra-high-pressure extraction process for GLT. (**B**) TIC of GLT using a targeted DMRM method for acidic compounds. Peaks in (**B**): 2. Ganoderic acid I; 3. Ganoderenic acid C; 4. Ganoderic acid C2; 5. Ganoderic acid C6; 6. Ganoderic acid G; 7. Ganoderenic B; 8. Ganoderic acid N; 9. Ganoderic acid B; 10. Ganoderic acid LM2; 11. Ganoderenic acid A; 12. Ganoderic acid K; 13. Ganoderenic acid E; 14. Ganoderic acid A; 15. Ganoderic acid H; 16. Lucidenic acid A; 17. Ganoderenic acid D; 18. Ganoderic acid D; 20. Ganoderic acid F; 22. Ganoderic acid DM; 25. Ganoderic acid Y. (**C**) TIC of GLT using a targeted DMRM method for neutral compounds. Peaks in (**C**): 1. 3,7,15-trihydroxy-11-oxo-lanosta-8-en-24-20 lactone; 2. Ganolactone B; 4. Ganoderlactone D; 5. 20-HydroxyGXG; 7. Ganodermanontriol; 8. Lucialdehyde B; 11. Ganoderiol F; 12. Ganodermanondiol; 13. Ganoderol B; 14. Lucidal; 15. Lucialdehyde A; 16. Ganoderol A; 17. Ganoderal A.

In this study, GLT was extracted using the ultrahigh-pressure extraction, with a schematic diagram of the process provided in Figure 1A. The total triterpenoid content in GLT was 302.4 mg/g, as determined using the vanillin-perchloric acid colorimetric method. Furthermore, the composition of GLT was identified using UPLC-QQQ-MS analysis as shown in Figure 1B,C: a total of 33 triterpenoids were identified in GLT, comprising 20 acidic and 13 neutral compounds. The 20 acidic triterpenoids were Ganoderic acid I, Ganoderenic acid C, Ganoderic acid C2, Ganoderic acid C6, Ganoderic acid G, Ganoderenic B, Ganoderic acid N, Ganoderic acid B, Ganoderic acid LM2, Ganoderenic acid A, Ganoderic acid K, Ganoderenic acid E, Ganoderic acid A, Ganoderic acid H, Lucidenic acid A, Ganoderenic acid D, Ganoderic acid D, Ganoderic acid F, Ganoderic acid DM, and Ganoderic acid Y (Figure 1B), and the 13 neutral triterpenoids were 3,7,15-trihydroxy-11-oxo-lanosta-8-en-24-20 lactone, Ganolactone B, Ganoderlactone D, 20-HydroxyGXG, Ganodermanontriol, Lucialdehyde B, Ganoderiol F, Ganodermanondiol, Ganoderol B, Lucidal, Lucialdehyde A, Ganoderol A, and Ganoderal A (Figure 1C). The data for the composition and contents of GLT are presented in Supplementary Table S1.

### 3.2. GLT Inhibits HFD-Induced Obesity and Fat Accumulation in C57BL/6J Mice

**Figure 2:**
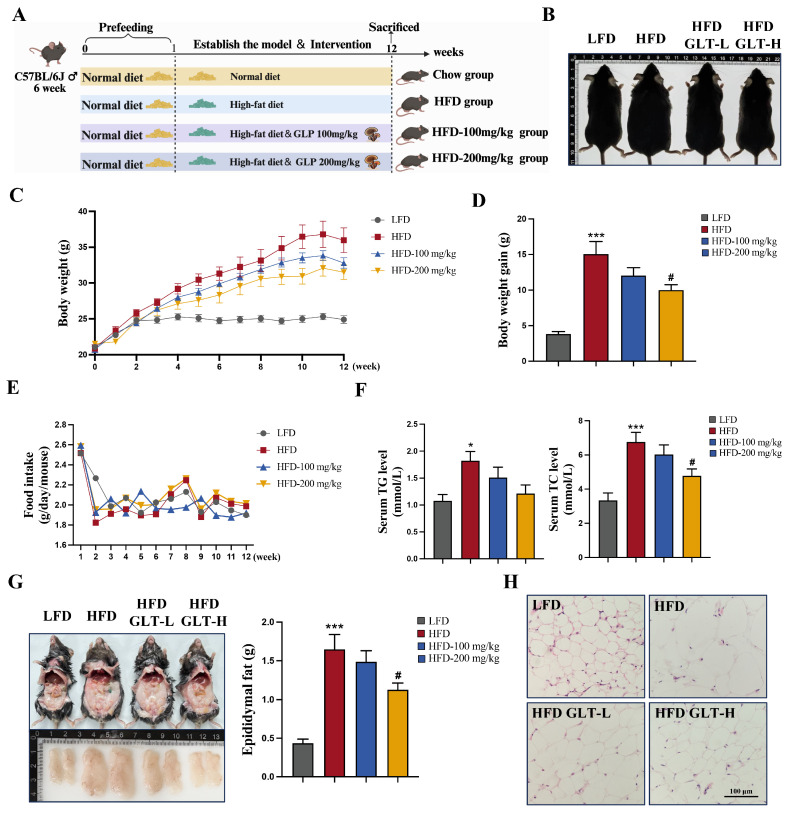
GLT attenuates weight gain and fat accumulation in HFD-fed mice. (**A**) Schematic illustration of the animal study design. (**B**) Representative images of mouse body size from each treatment group. (**C**) Continued body weight curve of mice after the treatment of GLT upon HFD for 12 weeks. (**D**) Body weight gain. (**E**) Food intake. (**F**) Levels of TG and TC in the serum of mice. (**G**) Representative images and weight measurement of eWAT accumulation in mice. (**H**) Representative H&E staining images of eWAT (scale bar, 100 μm). * *p* < 0.05, *** *p* < 0.001 as compared to LFD. ^#^ *p* < 0.05 as compared to HFD.

To investigate the role of GLT in obesity, we first examined the effects of GLT on body weight and lipid accumulation in HFD-fed mice. Figure 2A shows the schematic diagram of the experimental design for the animal study. As shown in Figure 2B, compared to controls, the body size of the mice significantly increased in the HFD group. Under an HFD, both doses of GLT significantly reduced body size compared to the HFD control alone (Figure 2B). After HFD feeding for four weeks, the body weight exceeded that of the of LFD group in mice fed a HFD, and this difference persisted until the end of the study (Figure 2C). However, the body weight was significantly reduced in both GLT group in a dose-dependent manner as compared to the HFD group (Figure 2C). The final weight gain demonstrated the same results (Figure 2D). In addition, we found that food intake did not differ significantly among all groups (Figure 2E). Similar water intake was observed across all groups. Compared with the LFD group, the TG and TC levels in serum were significantly higher in the HFD-fed mice, and this was reversed upon GLT treatment (Figure 2F).

It is well established that obesity is closely associated with the accumulation of lipids. We next examined whether GLT can inhibit fat accumulation in HFD-induced obese mice. As shown in Figure 2G, the HFD significantly increased the epididymal fat distribution and the weight of eWAT, while GLT at either dose significantly reduced fat accumulation in eWAT under the HFD (Figure 2G). In addition, H&E staining of eWAT showed that the size of adipocytes remarkably increased in the HFD group as compared to the LFD group, while the size of the eWAT was obviously reduced in HFD-fed mice treated with either dose of GLT (Figure 2H). Collectively, these results indicate that GLT inhibits body weight gain and ameliorates fat accumulation in the eWAT of obese mice.

### 3.3. GLT Inhibits Preadipocyte Differentiation and Lipid Accumulation in 3T3-L1 Cells

**Figure 3:**
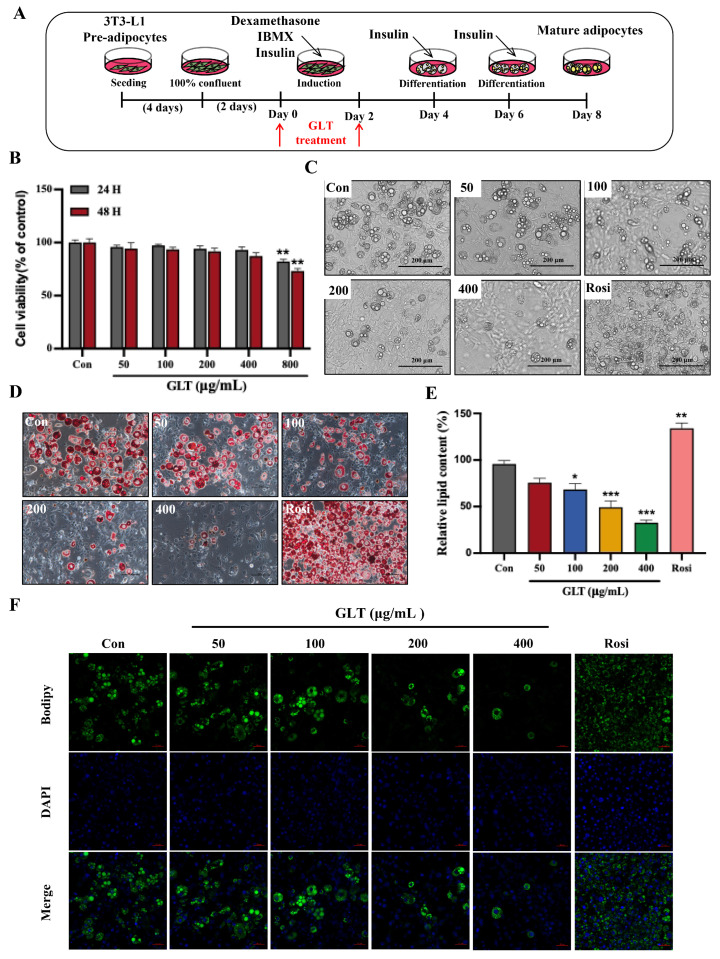
GLT inhibits preadipocyte differentiation and adipogenesis in 3T3-L1 cells. (**A**) Schematic diagram of cell experiment design. (**B**) Cell viability analysis. (**C**) Microscopic observation of 3T3-L1 cells at D8 of differentiation (scale bar, 200 μm). (**D**) Oil Red O staining of 3T3-L1 cells at D8 of differentiation (scale bar, 500 μm). (**E**) Quantitative analyses of Oil Red O staining. (**F**) Bodipy 490/503 fluorescent staining (red scale bar, 50 μm). * *p* < 0.05, ** *p* < 0.01, *** *p* < 0.001 as compared to Con.

It is known that preadipocyte differentiation is a key mechanism of adipogenesis and fat accumulation. Given our previous in vivo findings that GLT significantly attenuated fat deposition in obese mice, we next employed the 3T3-L1 preadipocyte model to investigate whether GLT inhibits lipid accumulation by suppressing preadipocytes differentiation. The schematic diagram of 3T3-L1 differentiation induction and GLT treatment is depicted in Figure 3A. We first determined non-cytotoxic doses of GLT in 3T3-L1 cells. The effects of various concentrations of GLT (50, 100, 200, 400 or 800 μg/mL) on the cell viability of 3T3-L1 preadipocytes were detected using an MTT assay. As shown in Figure 3B, GLT at concentrations up to 800 μg/mL induced significant cytotoxic effects (*p* < 0.01). Therefore, concentrations of GLT at 50, 100, 200, and 400 μg/mL were used in follow-up experiments. To investigate the role of GLT in adipogenesis and lipid accumulation, 3T3-L1 cells were treated with different concentrations of GLT and induced to differentiate for 8 days. Morphological observation showed that the preadipocytes differentiated gradually into round or circular-like mature adipocytes, and round lipid droplets appeared in the cytoplasm, whereas GLT treatment significantly inhibited adipogenic differentiation in a concentration-dependent manner (Figure 3C). Compared with the control cells, Oil Red O staining revealed that GLT significantly reduced the intensity of staining in a concentration-dependent manner (Figure 3D). The inhibitory effect of GLT on lipid droplet formation was verified after the quantification of Oil Red O staining (Figure 3E). In addition, the Bodipy 493/503 staining further confirmed that GLT markedly suppressed lipid droplet accumulation (Figure 3F). Rosiglitazone (Rosi) was used as a positive control and significantly promoted the adipogenic differentiation and lipid droplet accumulation (Figure 3C–E).

**Figure 4:**
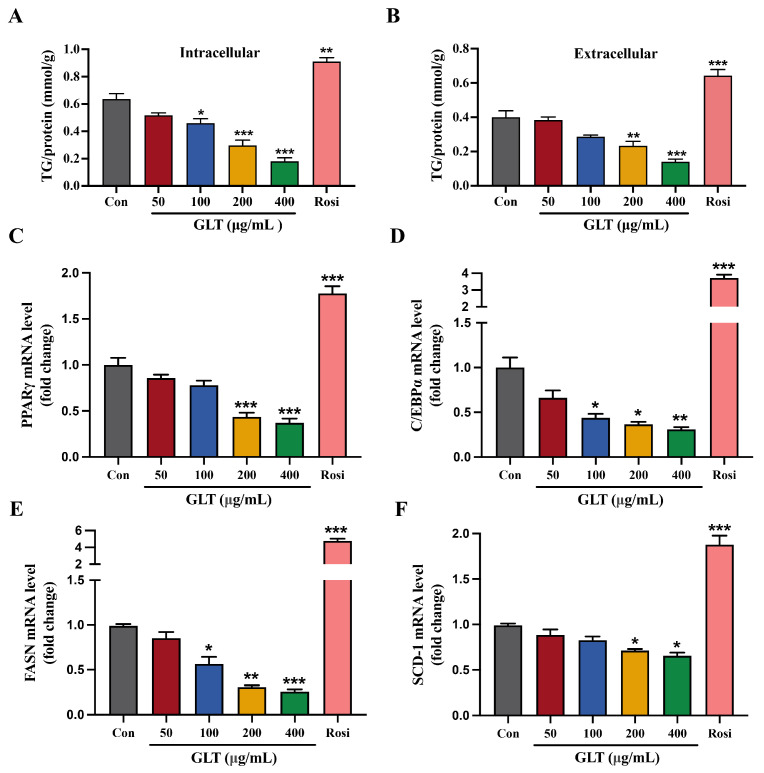
GLT inhibits lipid accumulation and the expression of adipogenesis-regulatory genes in 3T3-L1 cells. (**A**) Intracellular and extracellular (**B**) TG contents of mature 3T3-L1 adipocytes at D8 of differentiation. (**C**–**F**) qRT-PCR analysis of the mRNA expression of *PPARγ*, *C/EBPα*, *FASN*, and *SCD-1* at D8 of differentiation in 3T3-L1 cells. * *p* < 0.05, ** *p* < 0.01, *** *p* < 0.001 as compared to Con.

Furthermore, the levels of intracellular and extracellular TG were also quantified on day 8 of adipogenic differentiation. As shown in Figure 4A,B, GLT treatment concentration-dependent reduced both intracellular and extracellular TG levels in 3T3-L1 cells compared to control cells. We next examined whether GLT affects the expression of the most important adipogenic regulators, including *PPARγ*, *C/EBPα*, *FASN*, and *SCD-1*. As expected, the mRNA expression of these genes was significantly decreased upon GLT treatment in a concentration-dependent manner (Figure 4C–F). Taken together, these results indicate that GLT significantly inhibits adipogenic differentiation and lipid accumulation in 3T3-L1 preadipocytes.

### 3.4. Potential Targets and Pathways Analysis of GLT’s Inhibitory Effect on Adipogenesis

**Figure 5:**
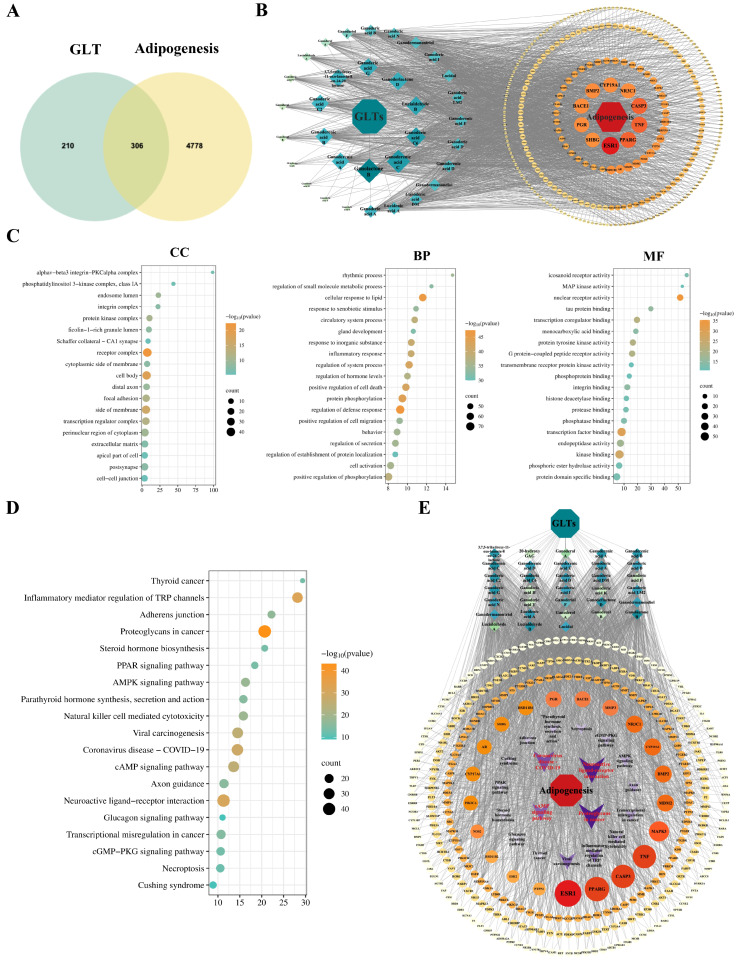
Network pharmacology analysis of Ganoderma lucidum triterpenoids in inhibiting adipocyte differentiation. (**A**) Venn diagram of the overlapping targets between GLT and adipocyte differentiation. (**B**) “GLT–active ingredient–target–adipogenesis” network. (**C**) Bubble maps of GO enrichment analysis. (**D**) Bubble maps of KEGG enrichment analysis. (**E**) Interaction network of “GLT–active ingredient–target pathway–adipogenesis”.

Network pharmacology was used to identify the potential targets and pathways of GLT in inhibiting adipogenesis. As shown in Figure 5A, the number of potential targets was 516, corresponding to 34 active ingredients obtained from Pharmmapper, Swisstarget Prediction, and the TCMSP database. For adipocyte-differentiation-related targets, 5084 targets were selected from the OMIM and Genecards databases (Figure 5A). A total of 306 common targets between GLT and adipocyte differentiation were acquired (Figure 5A), which were regarded as potential targets for GLT to regulate adipocyte differentiation. The interaction network of “GLT–active ingredient–target–adipogenesis” was constructed using Cytoscape 3.9.1, which included 342 nodes and 2284 edges (Figure 5B). Ganolactone B was associated with the highest number of targets, with 82 targets directly linked to it, suggesting that it may occupy a pivotal position in the network. In addition, ganoderic acid C6 and ganoderenic acid C also play prominent roles in this network, with each being connected to 79 targets.

To investigate the biological function of GLT in adipogenic differentiation, we performed GO enrichment analysis. As shown in Figure 5C, the interaction between GLT and adipogenesis was mainly involved in the following categories: regulation of receptor complex, protein kinase complex, transcription regulator complex, and other cellular components. GLT was highly relevant to lipid reaction (in line with Figure 3) and protein phosphorylation regulation, as well as other biological processes (Figure 5C). Its molecular functions mainly related to protein kinase activity, nuclear receptor activity, and transcription factor binding (in line with Figure 4) (Figure 5C). In addition, KEGG analysis showed that pathways related to lipid metabolism were enriched, including the AMPK signaling pathway, steroid hormone biosynthesis, and cAMP signaling pathway (Figure 5D). Moreover, consistent with Figure 4, genes of the PPAR signaling pathway were also enriched through KEGG analysis (Figure 5D). Additionally, the active ingredient–target pathway network showed that GLT acted with multiple targets and affected multiple pathways (Figure 5E).

### 3.5. Screening of Core Targets for GLT-Mediated Inhibition of Adipogenesis

**Figure 6:**
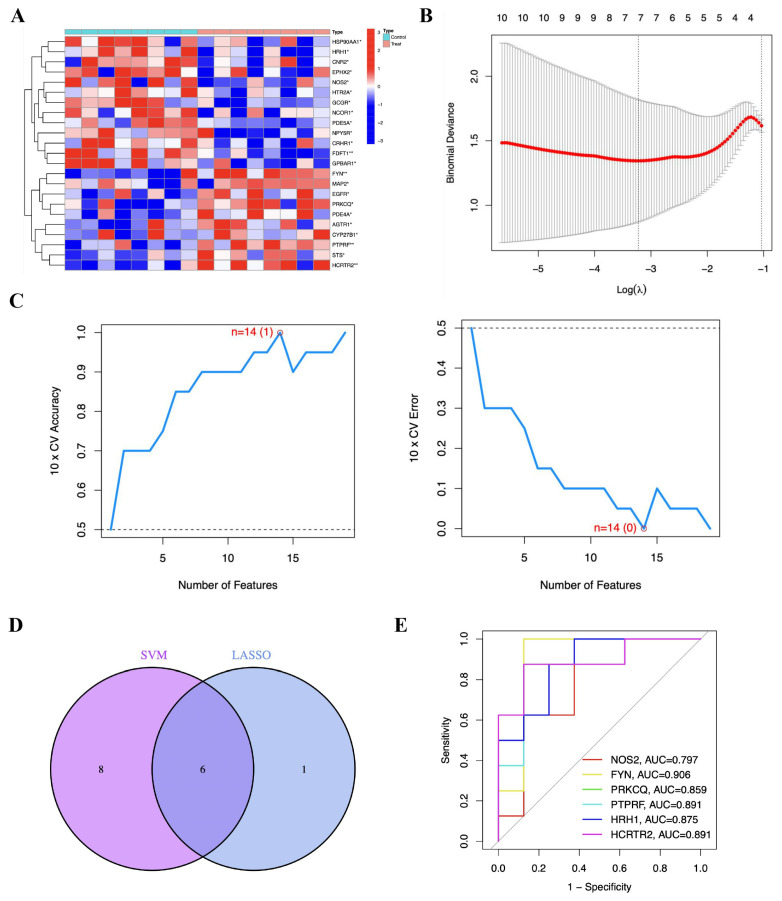
Analysis of core targets for GLT-mediated inhibition of adipogenesis. (**A**) Expression analysis of the predicted potential targets in obese patients using publicly available GEO database. (**B**) The parameter diagram obtained by LASSO algorithm. (**C**) Accuracy plot and cross-validation error plot of the SVM-RFE algorithm. (**D**) Venn diagram of overlapping targets between LASSO and SVM-RFE algorithms. (**E**) ROC curves of the predicted core targets for GLT-inhibited adipogenesis. * *p* < 0.05, ** *p* < 0.01 as compared to normal controls.

To further identify the core targets through which GLT inhibits adipogenesis, we performed gene expression analysis of the predicted potential targets in obese patients using the publicly available GEO database. As shown in Figure 6A, 23 of 306 predicted targets were significantly differentially expressed in visceral adipose tissue between obese patients and normal controls (*p* < 0.05). Compared with the normal control group, the expression of 10 prediction targets was significantly upregulated in the visceral adipose tissue of obese patients, including *EGFR*, *AGTR1*, *FYN*, *MAP2*, *CYP27B1*, *PRKCQ*, *PED4A*, *PTPRF*, *STS*, and *HCRTR2*. In addition, 13 predicted targets were significantly downregulated in the visceral adipose tissue of obese patients, including *NOS2*, *NCOR1*, *HSP90AA1*, *CNR2*, *HTR2A*, *PDE5A*, *CRHR1*, *FDFT1*, *HRH1*, *GPBAR1*, *GCGR*, *EPHX2*, and *NPY5R* (Figure 6A).

Next, we performed characteristic gene analysis on 23 differentially expressed genes using the LASSO regression and SVM-RFE algorithms. As shown in Figure 6B, the parameter corresponding to the dotted line on the left of the parameter diagram obtained by the LASSO algorithm was called lambda.min, and its corresponding parameter was 7; the seven characteristic genes were *NOS2*, *FYN*, *PRKCQ*, *PTPRF*, *FDFT1*, *HRH1*, and *HCRTR2*. Furthermore, the SVM-RFE algorithm was also applied to screen characteristic genes. As shown in Figure 6C, there are 14 genes identified with an accuracy of 1 and an error rate of 0, including *HRH1*, *PDE5A*, *PRKCQ*, *HCRTR2*, *NOS2*, *FYN*, *HSP90AA1*, *GPBAR1*, *EGFR*, *AGTR1*, *HTR2A*, *MAP2*, *PTPRF*, and *CRHR1*. The characteristic genes obtained from the LASSO and SVM-RFE models were cross-referenced, and Venn diagrams were applied to filter out six characteristic genes (*NOS2*, *FYN*, *PRKCQ*, *PTPRF*, *HRH1*, and *HCRTR2*) (Figure 6D).

To further screen the core targets, we constructed a logistic regression model of the six characteristic genes above and evaluated their diagnostic value in adipogenesis using the GSE24883 database. As shown in Figure 6E, ROC curves of these six characteristic genes were generated and the AUC were 0.797, 0.906, 0.859, 0.891, 0.875, and 0.891, respectively. It is well established that the AUC value range is between 0.5 and 1; the closer the AUC is to 1, the higher the diagnostic value [23]. Therefore, *FYN*, *PRKCQ*, *PTPRF*, *HRH1,* and *HCRTR2* (AUC > 0.85) were proposed as candidate core targets underlying the anti-adipogenic effect of GLT. In addition, we also found that the components regulating these core targets were primarily neutral triterpenoids, including Ganoderal A, Ganoderiol F, Ganoderlactone D, Ganodermanondiol, Ganodermanontriol, Lucialdehyde A, Lucialdehyde B, Lucidal, Ganolactone B, and 3,7,15-trihydroxy-11-oxo-lanosta-8-en-24-20 lactone.

### 3.6. GLT Inhibits Adipocyte Differentiation by Regulating PRKCQ

**Figure 7:**
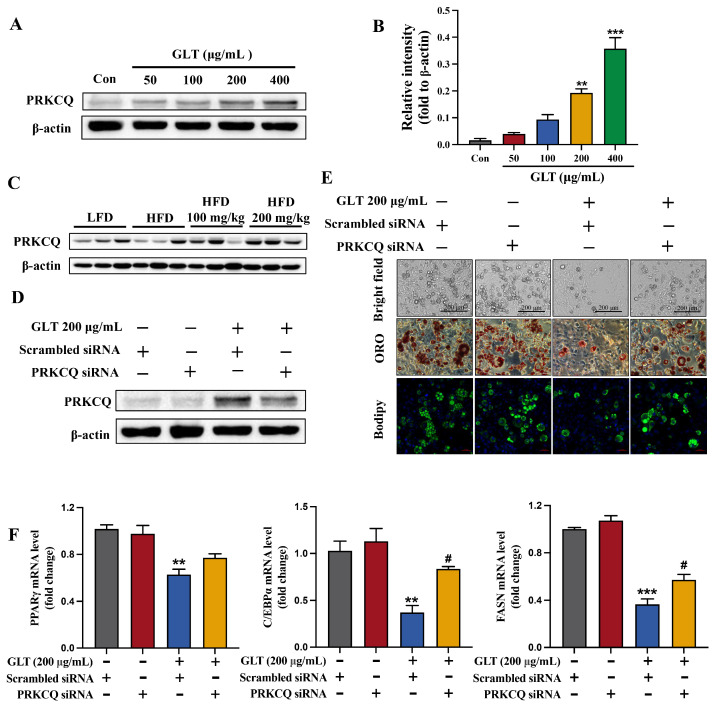
GLT inhibits adipogenesis and lipid accumulation by inducing PRKCQ. (**A**) Western blotting and densitometry analysis (**B**) of PRKCQ expression at protein level in 3T3-L1 cells at D8 of differentiation. (**C**) Western blotting analysis of the expression of PRKCQ in eWAT of HFD-fed mice. (**D**) Western blotting analysis of PRKCQ protein expression following PRKCQ siRNA treatment in 3T3-L1 cells. (**E**) Analysis of cell morphology (scale bar, 200 μm), Oil Red O (scale bar, 500 μm), and Bodipy 490/503 staining (red scale bar, 50 μm) in 3T3-L1 cells treated with GLT and PRKCQ siRNA on D8 of differentiation. (**F**) qRT-PCR analysis of the mRNA expression of *PPARγ*, *C/EBPα*, *FASN*, and *SCD-1* in 3T3-L1 cells treated with GLT and PRKCQ siRNA on D8 of differentiation. ** *p* < 0.01, *** *p* < 0.001 as compared to Con or Group treated with Scrambled siRNA alone. ^#^ *p* < 0.05 as compared to Group treated with GLT and Scrambled siRNA.

Based on the results from Figure 5E, *PRKCQ* and *HCRTR2* were found to be targeted by a greater number of GLT components, suggesting they may serve as the key targets mediating the effects of GLT. Furthermore, *PRKCQ* has been reported to play an important role in adipocyte differentiation, while *HCRTR2* is associated with the browning of adipose tissue [24,25]. Therefore, we next focused on validating the role of PRKCQ in the suppression of adipocyte differentiation by GLT. First, we examined the effect of GLT on the expression of PRKCQ during adipocyte differentiation. As shown in Figure 7A,B, Western blotting results showed that GLT significantly induced PRKCQ protein expression in a concentration-dependent manner at day 8 after differentiation in 3T3-L1 cells. Furthermore, the expression of PRKCQ was obviously reduced in the eWAT of HFD-fed mice compared to the controls (Figure 7C). However, GLT significantly increased PRKCQ expression in the eWAT of obese mice (Figure 7C). Consistent with our previous bioinformatic analysis, the expression of PRKCQ exhibited significant changes upon GLT treatment, further suggesting that PRKCQ may be a key target in regulating adipogenesis. Our data suggest that GLT could inhibit adipogenic differentiation by upregulating the expression of PRKCQ.

We further examined whether PRKCQ plays a role in the GLT-induced inhibition of adipocyte differentiation. We treated 3T3-L1 cells with PRKCQ siRNA to knock down *PRKCQ*. As shown in Figure 7D, the expression of PRKCQ was significantly reduced upon PRKCQ siRNA treatment. 3T3-L1 cells were then differentiated for 8 days with or without GLT treatment. Using the white light image, Oil Red O staining, and Bodipy staining, we observed that the knockdown of *PRKCQ* significantly reversed (at least partially) the inhibition of GLT on adipocyte differentiation (Figure 7E). Furthermore, the inhibitory effect of GLT on the expression of *PPARγ*, *C/EBPα*, and *FASN* was also significantly reduced after PRKCQ elimination (Figure 7F). Taken together, these results indicate that GLT inhibits adipocyte differentiation and lipid accumulation by targeting PRKCQ.

## 4. Discussion

Obesity is an urgent public health problem defined by adipose tissue hypertrophy, often accompanied by different degrees of inflammation and various chronic metabolic diseases [26,27]. However, the management of obesity remains a significant challenge, owing to the limited variety of currently available anti-obesity drugs and their associated adverse effects [28,29]. The inhibition of adipogenesis represents a key target in the prevention of obesity [26]. Evidence indicates that natural products contain bioactive components, such as quinoa [30], Paliurus ramosissimus [31], ginger [32], and *G. lucidum* [33], which suppress obesity by regulating adipogenic differentiation and adipogenesis. Despite previous reports on the anti-obesity effects of *G. lucidum* as a medicinal and edible substance, research on its triterpenoid extract remains limited and the underlying mechanisms are still unclear. In the current study, we found that *G. lucidum* triterpenoid extract inhibited obesity and fat accumulation in HFD-fed obese mice, which may be associated with the regulation of adipogenesis in the adipose tissue. Furthermore, we demonstrated for the first time that GLT inhibited adipocytes differentiation and lipid accumulation by activating PRKCQ. Our study provides a theoretical basis for the future development of GLT as a pharmaceutical agent or functional food for preventing obesity and its related metabolic disorders.

To date, several laboratory studies have reported the anti-obesity activity of GLT. In an early study, Lee et al. [34,35,36] systematically identified several lanostane triterpenes from *G. lucidum* fruiting bodies that inhibit adipogenesis in 3T3-L1 cells. Their key mechanistic insight was that these active compounds, notably specific butyl ester derivatives, suppressed adipocyte differentiation and lipid accumulation by downregulating master adipogenic regulators, including *PPARγ*, *C/EBPα*, and *SREBP-1c*, along with their target lipogenic enzymes. Another early study reported [37] that Methyl Ganoderate extracted from *G. lucidum* significantly inhibited both the proliferation and differentiation of 3T3-L1 preadipocytes. Furthermore, treatment with this compound led to a marked reduction in intracellular triglyceride and total cholesterol levels [37]. In studies by Guo et al., research progressed from a triterpenoid-enriched ethanol extract of *G. lucidum* (GL95) to a purified constituent, ganoderic acid A (GA) [38]. Their work demonstrated that these interventions effectively alleviated high-fat-diet-induced hyperlipidemia and hepatic steatosis in rodent models. A key mechanistic insight was that the lipid-lowering effects were associated with modulation of the gut microbiota composition and regulation of host metabolic pathways involved in fatty acid and bile acid metabolism [38]. Recently, Tong et al. [39] reported that a triterpenoid extract from *G. lucidum* (GP) improved serum lipid profiles in hyperlipidemic rats. Notably, this effect was accompanied by the modulation of gut microbiota, specifically an increased abundance of Alloprevotella and a decreased proportion of Blautia. These studies suggest that GLT could inhibit obesity and lipid accumulation. Consistent with prior reports, our data further confirm that GLT effectively suppresses adipocyte differentiation and concentration-dependently diminishes lipid accumulation, contributing to its anti-obesity effects. Although these effects of GLT have been established, the specific targets and molecular mechanisms are still unknown.

Systems pharmacology has been utilized in several studies, especially within traditional Chinese medicine research, for the prediction of targets and mechanistic investigation. Li et al. [40] employed network pharmacology to reveal the multi-target mechanism through which mulberry leaves ameliorate obesity, involving regulation of the PI3K/Akt/Bcl-xl signaling pathway. Similarly, a network pharmacology study demonstrated the lipid-lowering effect of Si-Ni-SAN, with the activation of the AMPK signaling pathway implicated in promoting lipolysis for obesity intervention [41]. Using a network pharmacology approach, we identified 306 potential targets of GLT against adipogenic differentiation and constructed a “GLT–active ingredient–target–adipogenesis” network, which facilitates the subsequent establishment of linkage between key targets and anti-obesity effects of the GLT. In addition, signal pathway prediction revealed that GLT inhibition of adipogenic differentiation and adipogenesis may be associated with the PPAR, AMPK, and cAMP signaling pathways. It is well established that the PPAR signaling pathway plays an important role in adipogenic differentiation and adipogenesis [42,43]. Inhibition of the PPAR signaling pathway has been demonstrated as a key mechanism for suppressing adipogenesis [44,45]. Our study also found that GLT significantly inhibited the expression of PPARγ during 3T3-L1 preadipocytes differentiation. In addition, accumulating evidence suggests that activation of the AMPK signaling pathway plays an important role in inhibiting adipogenesis [11,46]. Unfortunately, this pathway was not verified in the present study, which should be further examined in future studies.

Transcriptomic data are characterized by high throughput, low cost, and precise quantification; when integrated with bioinformatics analyses, this allows for the efficient identification of key targets [47,48]. Machine learning algorithms, a class of algorithms that learn and acquire knowledge through human learning behaviors, have been extensively used in network pharmacology for prediction and classification by evaluating metrics such as precision, recall, and ROC [49,50]. Similarly, in our study, the core targets were analyzed based on the GSE24883 gene chip from the GEO database, combined with the LASSO regression and SVM-RFE algorithms. The final candidate core targets were identified as *PRKCQ*, *HCRTR2*, *PTPRF*, *FYN*, and *HRH1*. These targets are reportedly associated with the regulation of lipid metabolism and anti-obesity activity [51,52,53,54,55]. Our analysis revealed that *PRKCQ* and *HCRTR2* emerged as prominent targets within the network, potentially mediating the anti-adipogenic effects of GLT. Thus, our research was centered on these two specific targets.

*PRKCQ*, a gene encoding protein kinase C theta (PKCθ), is a member of the protein kinase C (PKC) family of lipid-dependent serine/threonine kinases activated by calcium ions (Ca^2+^) and diacylglycerol (DAG) [56]. Growing evidence indicates that PRKCQ is notably associated with the improvement of obesity. An early study reported a significant reduction in PRKCQ protein content in skeletal muscle from obese, insulin-resistant patients, suggesting that its altered regulation is a feature of the obese metabolic state. While the exact role appears complex, this foundational observation supports the broader premise that PRKCQ is a key regulator involved in obesity pathogenesis [57]. Another early study showed that *PRKCQ* knockout mice exhibited reduced energy expenditure and physical activity, leading to significantly increased adiposity and severe systemic insulin resistance when challenged with a high-fat diet. This genetic functional study provides direct in vivo evidence for the protective role of PRKCQ against obesity [58]. SERRA et al. [59] built genetic knockout models and found that transgenic mice expressing a dominant-negative PRKCQ mutant specifically in skeletal muscle developed age-dependent obesity and insulin resistance. In addition, Sun et al. reported that PRKCQ negatively regulates adipogenic differentiation and lipogenesis in 3T3-L1 preadipocytes, while activated PRKCQ reduces *PPARγ2* mRNA expression through activation of ERK signaling [51]. In the past debate, PKC enhances the stimulatory effects of adipogenesis under conditions of obesity, as obesity leads to oxidative stress in adipose tissue, which induces PKC regulation that enhances lipid storage and reduces mitochondrial uptake [51]. These studies suggest that PRCKQ could negatively regulate adipocyte differentiation and lipogenesis effectively. Consistent with the above results, we found that GLT treatment increased the expression of PRKCQ in the eWAT of HFD-fed obese mice and inhibited fat accumulation and obesity in mice fed a high-fat diet. In line with in vivo observations, our data showed that GLT significantly induced the expression of PRKCQ during 3T3-L1 preadipocytes differentiation. Furthermore, the inhibitory effect of GLT on adipogenesis was markedly reversed upon *PRKCQ* knockdown. Our findings demonstrate that GLT inhibits adipogenesis and lipid accumulation through the activation of PRKCQ.

The *HCRTR2* gene encodes a G-protein-coupled receptor that mediates regulation of feeding behavior. An early study reported that activation of the HCRTR2 pathway could combat diet-induced metabolic dysfunction by increasing energy expenditure and improving leptin sensitivity, positioning it as a potential therapeutic target for obesity and related disorders [60]. Kakizaki et al. investigated the metabolic effects of two different orexins on mice fed a high-fat diet. They demonstrated that HCRTR2 positively regulates energy expenditure in mice [61]. In another study, HCRTR2 was found to reduce hepatic gluconeogenesis, improving insulin sensitivity and glucose tolerance in obese mice and modulating peripheral glucose metabolism in obese patients [25]. Recently, HCRTR2 has been found to regulate sleep architecture and glucose metabolism. Loss of HCRTR2 function in melanin-concentrating hormone neurons disrupted non-rapid eye movement (NREM)-to-REM sleep transitions and promoted insulin resistance with compensatory hyperphagia, suggesting its role in linking sleep and metabolic physiology [62]. This integrated dysfunction suggests that impaired HCRTR2 signaling in specific neural populations may represent a novel pathway contributing to obesity risk by concurrently disrupting sleep quality and metabolic homeostasis. Therefore, the anti-obesity effect of HCRTR2 may be mediated through the regulation of energy metabolism and insulin sensitivity. Due to the limitations of the current model, the role of HCRTR2 in the anti-obesity effects of GLT will be verified in future studies.

Furthermore, our results showed that the components of GLT that regulate core targets are primarily neutral triterpenoids, including Ganoderal A, Ganoderiol F, Ganoderlactone D, Ganodermanondiol, Ganodermanontriol, Lucialdehyde A, Lucialdehyde B, Lucidal, Ganolactone B and 3,7,15-trihydroxy -11-oxo-lanosta-8-en-24-20 lactone, suggesting that neutral triterpenoids may exhibit more potent anti-obesity activity. However, the existing research has largely concentrated on acidic GLT in combating obesity, with little attention paid to the potential role of neutral GLT in combating obesity, for instance, Resinacein S [63], Methyl Ganoderate [37], and ganoderic acid A [64]. Interestingly, in Li et al.’s study on the anti-tumor effects of GLT, neutral GLT exhibited a more potent inhibitory effect on cancer cell proliferation than acidic GLT [65]. Thus, we speculate that neutral GLT could be pivotal in anti-obesity interventions. In future studies, we will isolate both neutral and acidic GLT fractions and conduct a direct comparison of their anti-obesity efficacy.

## 5. Conclusions

**Figure 8:**
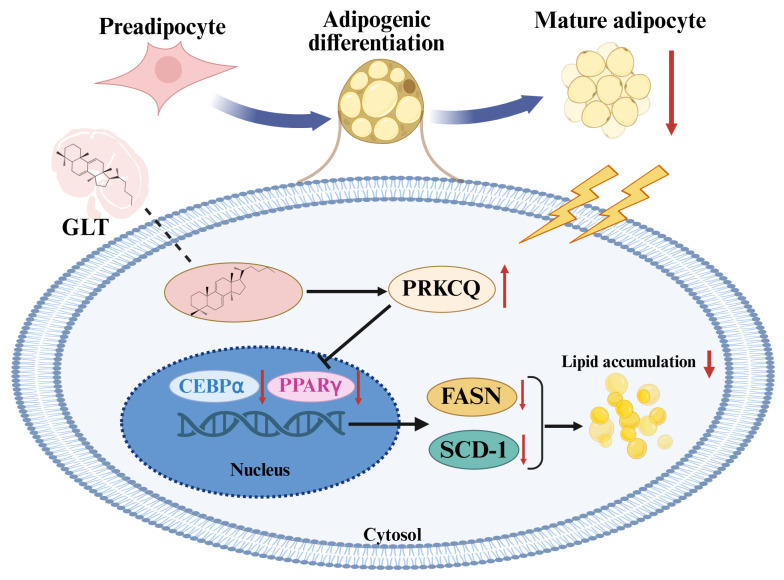
Schematic showing the molecular mechanisms elicited by GLT in inhibiting adipogensis.

In conclusion, as shown in Figure 8, our results suggest that triterpenoids extracted from *G. lucidum* suppress adipogenesis and lipid accumulation by inducing PRKCQ expression, which in turn downregulates the key adipogenic transcription factors *PPARγ* and *C/EBPα*, leading to reduced expression of the lipogenic genes *FASN* and *SCD-1*. In addition, GLT treatment inhibited obesity and fat accumulation in HFD-fed obese mice. Owing to limitations of the model system, this study did not explore additional targets or pathways underlying the anti-obesity effects of GLT. Sustained investigation employing diverse obesity models and clinical adipose tissue samples will help to further elucidate the molecular mechanisms by which GLT inhibits adipogenesis and obesity. This study provides novel insights into the anti-obesity bioactivity of GLT and lays a solid theoretical foundation for the future development of anti-obesity pharmaceuticals and functional foods.

## Data Availability

The original contributions presented in this study are included in the article/Supplementary Materials. Further inquiries can be directed to the corresponding authors.

## Supplementary Materials

The following supporting information can be downloaded at https://www.mdpi.com/article/10.3390/foods15020325/s1: Table S1: Composition and contents of GLT (mg/g); File S1: The uncropped blot of Western blotting in this article.

## Abbreviations

The following abbreviations are used in this manuscript:

*G. lucidum*	*Ganoderma lucidum*
GLT	*G. lucidum* triterpenoids
FYN	FYN proto-oncogene, Src family tyrosine kinase
PRKCQ	protein kinase C theta
PTPRF	protein tyrosine phosphatase receptor type F
HRH1	histamine receptor H1
CRHR1	corticotropin releasing hormone receptor 1
HCRTR2	hypocretin receptor 2
TCM	traditional Chinese medicine
PPARγ	peroxisome proliferator-activated receptor gamma
C/EBPα	CCAAT/enhancer binding protein (C/EBP), alpha
FASN	fatty acid synthase
SCD-1	stearoyl-CoA Desaturase 1
UPLC-QQQ-MS/MS	ultra-Performance Liquid Chromatography–Triple Quadrupole Tandem Mass Spectrometry
DMEM	Dulbecco’s modified Eagle’s medium
IBMX	3-isobutyl-1-methylxanthine
DAPI	4′,6-diamidino-2-phenylindole
TG	the triglyceride
BCA	bicinchoninic acid
LASSO	least absolute shrinkage and selection operator
SVM-RFE	support vector machine–recursive feature elimination
DMI	differentiation medium I
DMII	differentiation medium II
DMSO	dimethyl sulfoxide
PBS	phosphate-buffered saline
SDS-PAGE	sodium dodecyl sulfate–polyacrylamide gel electrophoresis
PVDF	polyvinylidene fluoride
siRNA	small interfering RNAs
EGFR	epidermal growth factor receptor
AGTR1	angiotensin II receptor type 1
MAP2	microtubule associated protein 2
CYP27B1	cytochrome P450 family 27 subfamily B member 1
PED4A	phosphodiesterase 4A
STS	steroid sulfatase
NOS2	nitric oxide synthase 2
NCOR1	nuclear receptor corepressor 1
HSP90AA1	heat shock protein 90 alpha family class A member 1
CNR2	cannabinoid receptor 2
HTR2A	5-hydroxytryptamine receptor 2A
PDE5A	phosphodiesterase 5A
FDFT1	farnesyl-diphosphate farnesyltransferase 1
GPBAR1	G protein-coupled bile acid receptor 1
GCGR	glucagon receptor
EPHX2	epoxide hydrolase 2
NPY5R	neuropeptide Y receptor Y5
GO	gene ontology
KEGG	Kyoto Encyclopedia of Genes and Genomes
