# Supplementary material for: Ganoderma lucidum Triterpenoids Suppress Adipogenesis and Obesity via PRKCQ Activation: An Integrated In Vivo, In Vitro, and Systems Pharmacology Study
Source: Foods. 2026 Jan 15;15(2):325. doi: 10.3390/foods15020325 (PMC12841367; doi:10.3390/foods15020325)

The uncropped blot of Western blotting in this article.
Figure 7A:
PRKCQ

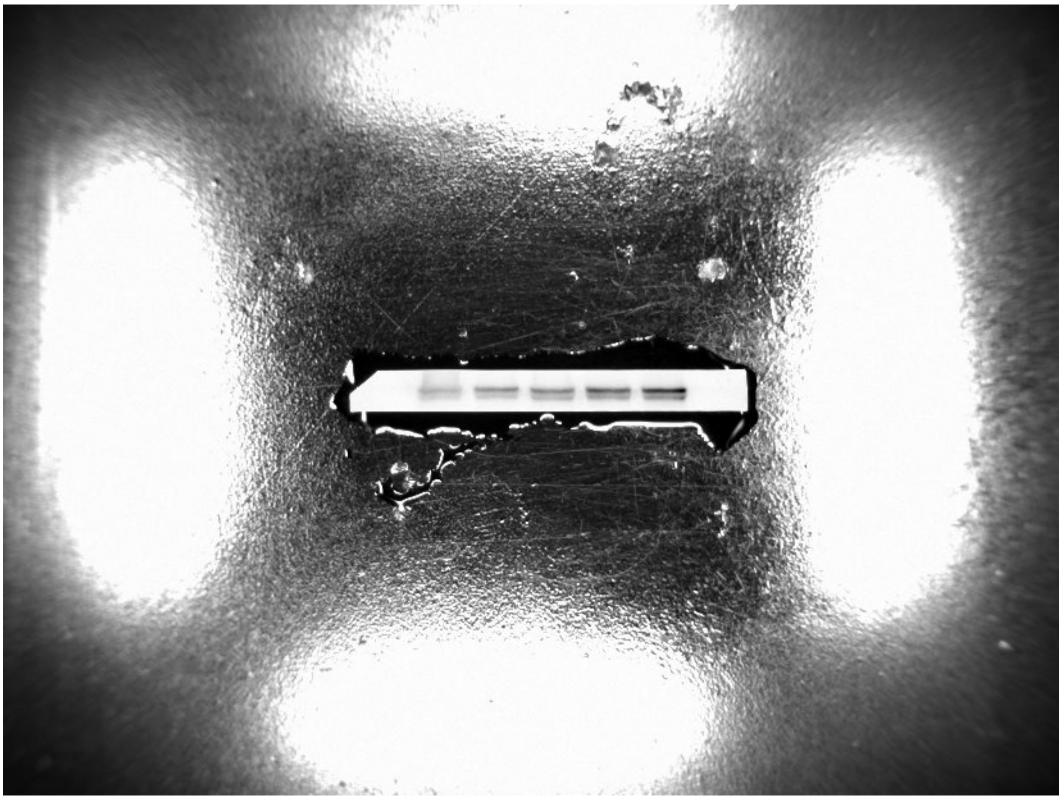

β-actin:

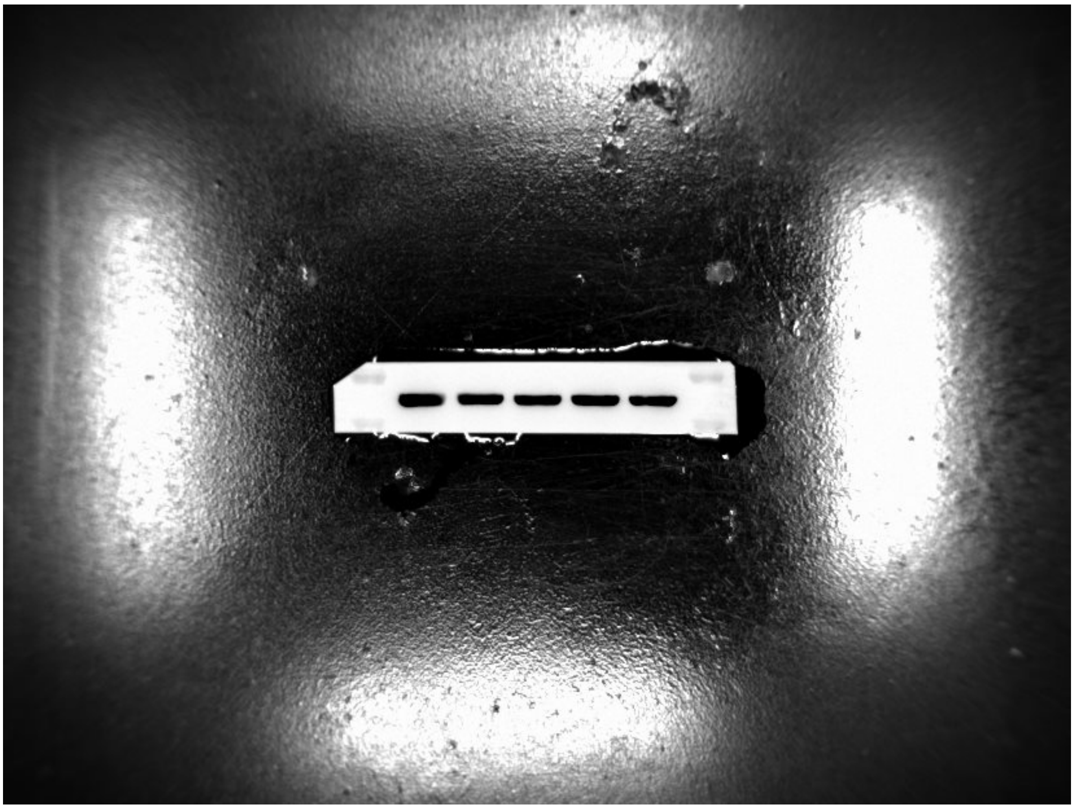

Figure 7C:
PRKCQ:

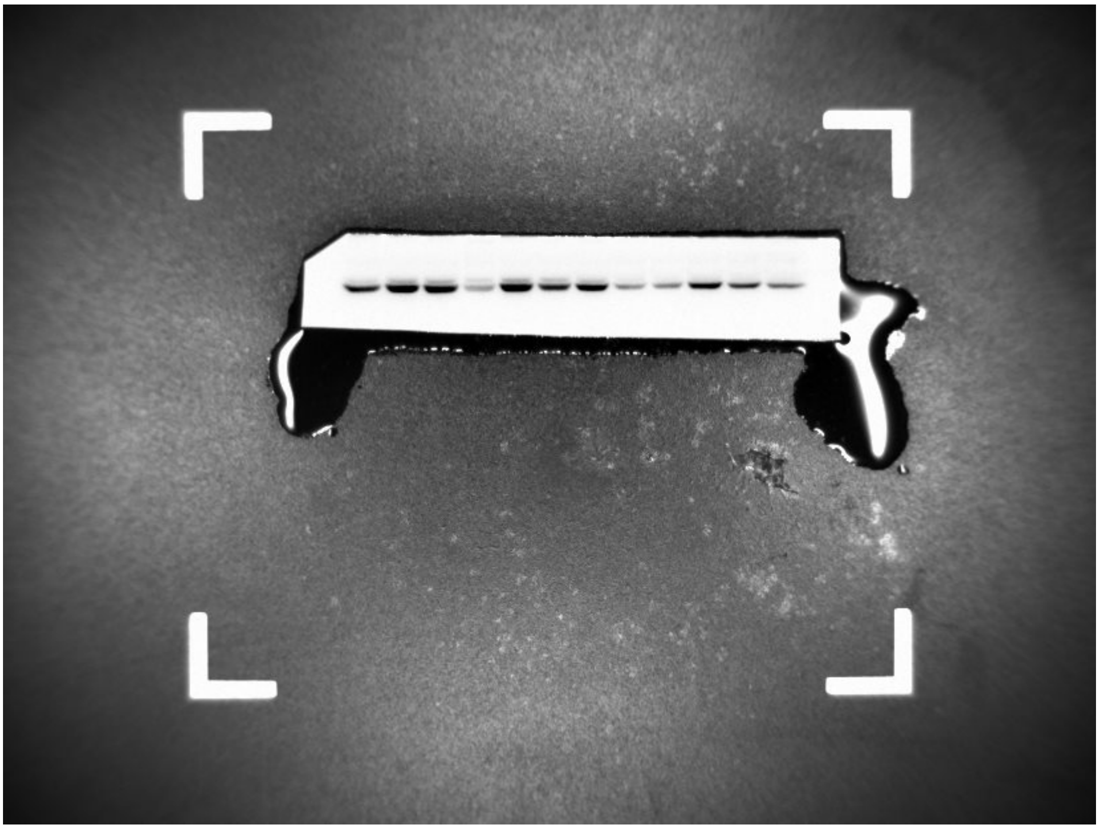

β-actin:

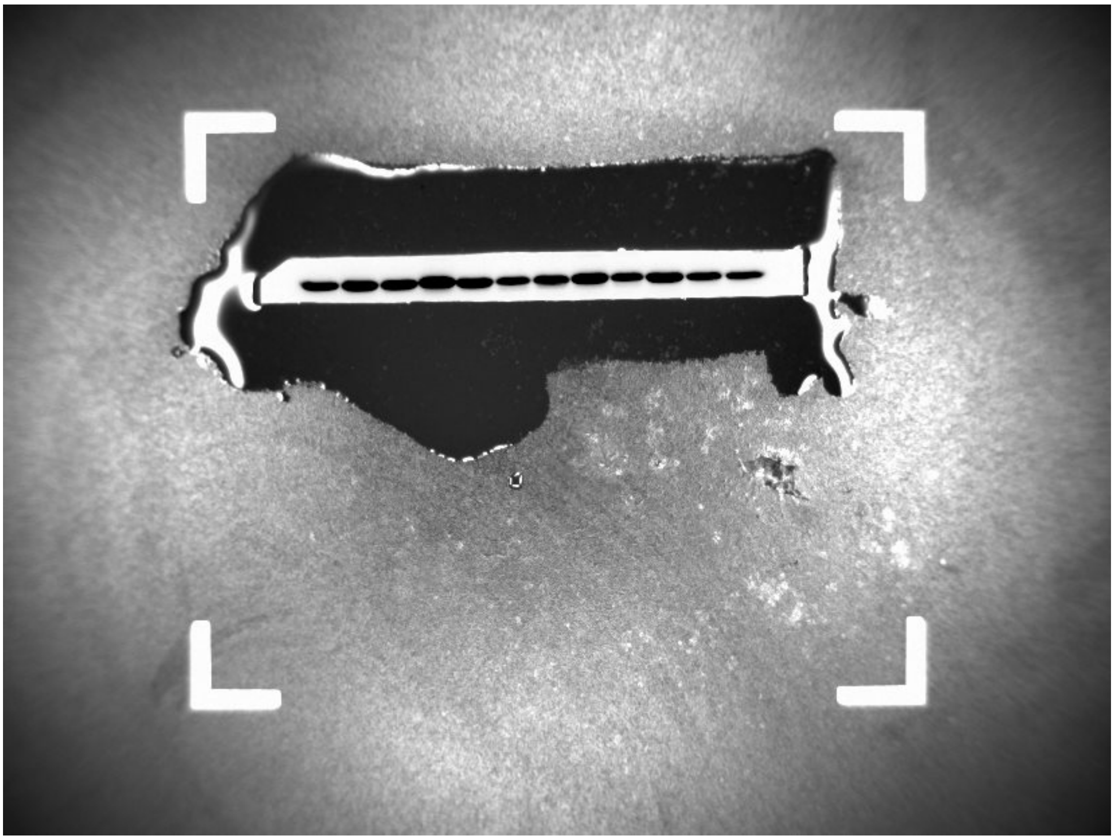

Figure 7D:
PRKCQ:

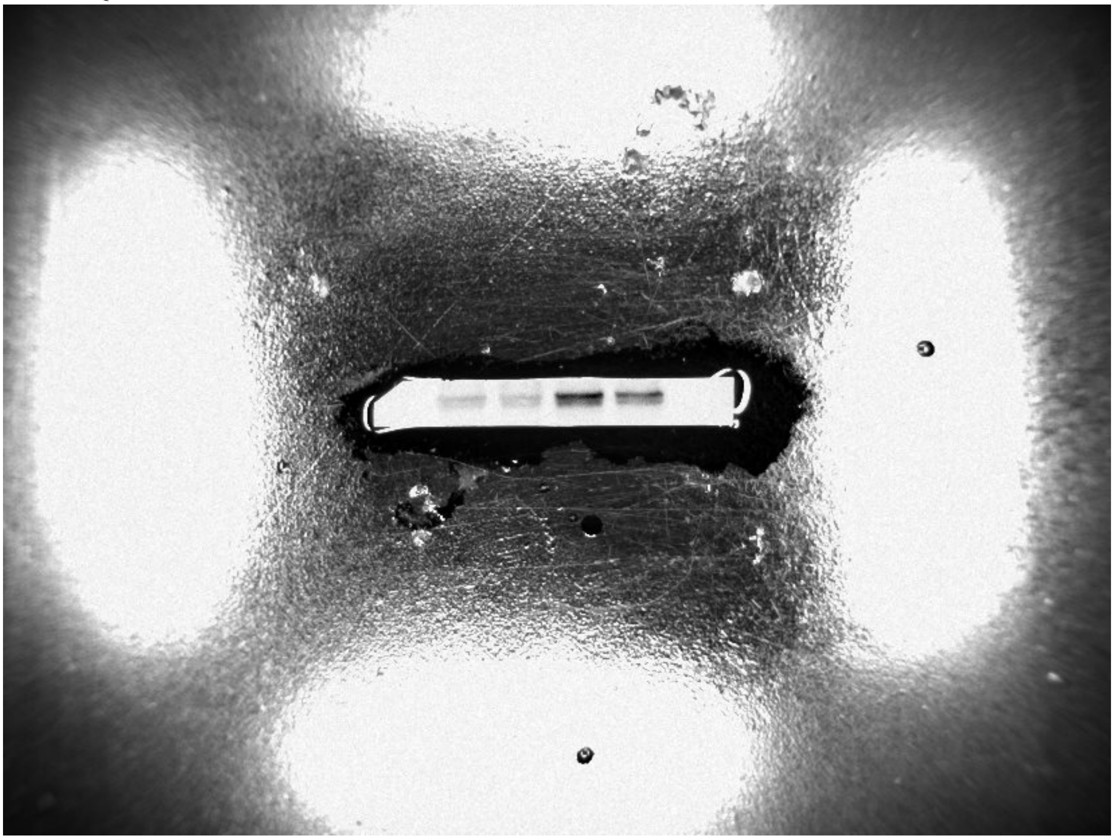

β-actin:

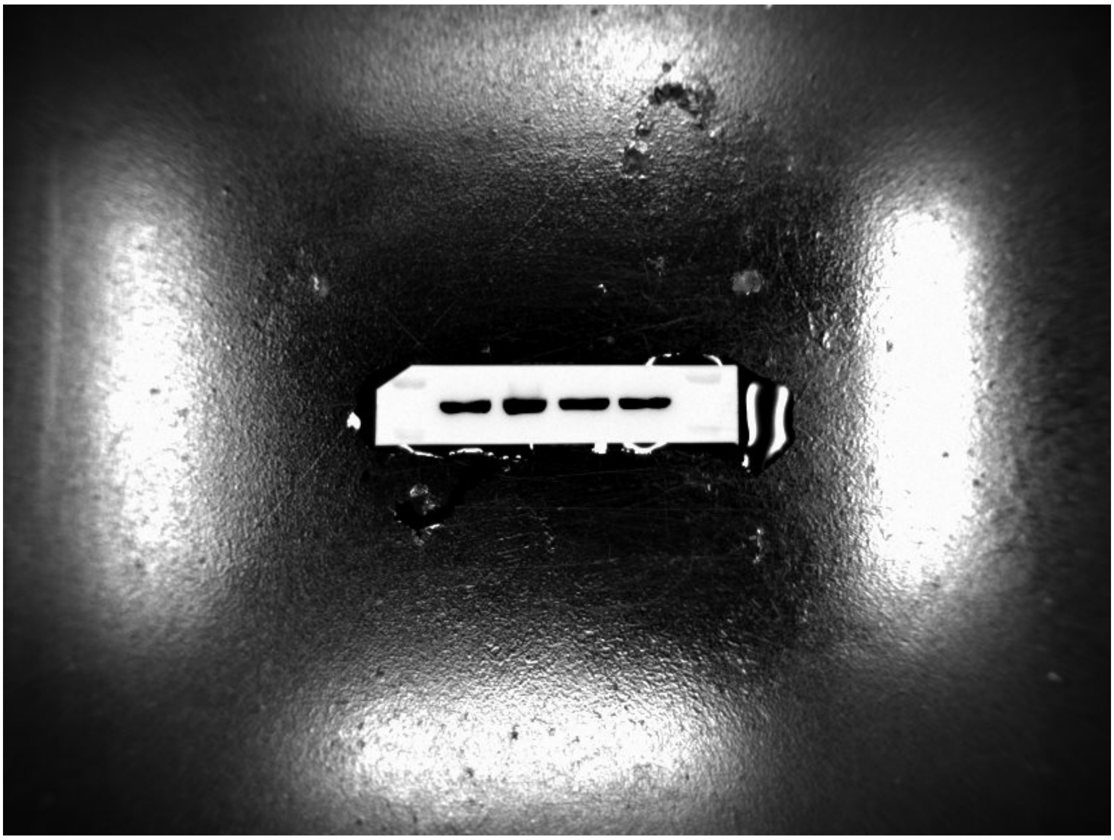

Supplement: Supplementary file 1 [file foods-15-00325-s001.zip › File S1.pdf]
