# Supplementary material for: Ganoderma lucidum Triterpenoids Suppress Adipogenesis and Obesity via PRKCQ Activation: An Integrated In Vivo, In Vitro, and Systems Pharmacology Study
Source: Foods. 2026 Jan 15;15(2):325. doi: 10.3390/foods15020325 (PMC12841367; doi:10.3390/foods15020325)
Supplement: Supplementary file 1 [file foods-15-00325-s001.zip › Table S1.pdf]

Supplementary Materials

Table S1. Composition and contents of GLT (mg/g)

The contents of 20 acidic triterpenes				
Ganoderic acid I	Ganoderenic acid C	Ganoderic acid C2	Ganoderic acid C6	Ganoderic acid G
2.8874	4.0370	4..4133	3.8500	9.4004
Ganoderenic B	Ganoderic acid N	Ganoderic acid B	Ganoderic acid LM2	Ganoderenic acid A
16.2777	4.2859	12.6850	7.3577	15.5960
Ganoderic acid K	Ganoderenic acid E	Ganoderic acid A	Ganoderic acid H	Lucidenic acid A
12.2813	1.0757	33.4998	14.4153	6.9435
Ganoderenic acid D	Ganoderic acid D	Ganoderic acid F	Ganoderic acid DM	Ganoderic acid Y
13.8914	16.2118	20.3479	5.6717	1.7622
The contents of 13 neutral triterpenes				
3,7,15-trihydroxy-11-oxo-lanosta-8-en-24-20 lactone	Ganolactone B	Ganoderlactone D	20-HydroxyGXG	Ganodermanontriol
0.0174	0.0527	0.0267	3.8447	8.4356
Lucialdehyde B	Ganoderiol F	Ganodermanondiol	Ganoderol B	Lucidal
1.9339	5.0169	2.3202	1.9548	0.0423
Lucialdehyde A	Ganoderol A	Ganoderal A		
1.2291	1.2009	5.0302		
